# Insights into Pyrroloquinoline Quinone (PQQ) Effects on Soil Nutrients and Pathogens from Pepper Monocropping Soil under Anaerobic and Aerobic Conditions

**DOI:** 10.1128/spectrum.00933-22

**Published:** 2022-07-19

**Authors:** Xin Li, Mingxing Zhang, Qingzhuang Zhang, Fangjun Tan, Zheng Gong, Yunhe Xie, Yu Tao, Jie Chen

**Affiliations:** a Hunan Academy of Agricultural Sciences, Changsha, Hunan, China; b Hunan Institute of Agricultural Environment and Ecology, Changsha, Hunan, China; c Hunan Vegetables Research Institute, Changsha, Hunan, China; d Key Laboratory for Agro-Environment in Midstream of Yangtze Plain, Ministry of Agriculture, Changsha, P.R. China; e Hunan Key Laboratory of Agro-Farmland Heavy Metal Pollution Control and Remediation, Hunan, China; USDA - San Joaquin Valley Agricultural Sciences Center

**Keywords:** pyrroloquinoline quinone, soil available nutrients, soil pathogens, microbial community

## Abstract

Imbalances of soil available nutrients and soilborne diseases have seriously restricted the productivity of crops and jeopardized food security worldwide. Pyrroloquinoline quinone (PQQ), a redox cofactor in some bacteria involved in glucose metabolism and phosphorus mineralization, could be anticipated to alter soil ecosystems to a certain extent. However, there is limited information on PQQ defending soilborne pathogens and regulating soil main nutrients. Here, a pot experiment based on mono-cropping soils of pepper was conducted to examine the effects of PQQ amendment on reconstructing soil microbial communities and soil nutrients under aerobic/anaerobic conditions comprising three treatments, namely, control, PQQ (aerobic), and FL-PQQ (anaerobic). The results revealed that soil microbial community composition and soil nutrients were distinctly altered by PQQ regimes. Compared to control, PQQ treatment significantly increased the content of soil available phosphorus (AP), while FL_PQQ treatment strongly improved the content of soil available nitrogen (AN). In terms of pathogens, relative to control, both PQQ treatments suppressed the abundances of pathogens, of which FL_PQQ treatment significantly decreased the abundance of the pathotrophic fungal by 64% and the abundance of Fusarium oxysporum by 57%, largely attributed to the increase of organic acid generators (O*xobacter*, *Hydrogenispora*) and potential antagonists (*Bacillus, Talaromyces*). Structural equation modeling (SEM) showed that PQQ regimes suppressed pathogens by indirectly regulating soil physicochemical properties and microbial communities. Overall, we proposed that PQQ application both in aerobic/anaerobic conditions could improve soil available nutrients and suppress soil pathogens in pepper monocropping soils.

**IMPORTANCE** The attention to PQQ (pyrroloquinoline quinone) effect on soil nutrients and pathogens was less paid in monocropping soils. However, the underlying microbial interacting mechanism remains unclear. Adopting a novel external bio-additive, the effects of PQQ on soil main nutrients and the pathotrophic fungal under aerobic and anaerobic regimes will be investigated, which would help to improve soil quality health. Our main conclusion was that PQQ would help to remediate monocropping obstacle soils in terms of soil nutrients and soil pathogens by associating with the microbial community, and anaerobic PQQ application more favored amelioration of continuous obstacle soils. These results will benefit the health and sustainable development of pepper production as well as other greenhouse vegetable production.

## INTRODUCTION

Vegetables are considered a source of human crucial nutrients and for the reduction of disease risks. As one of the largest producers and consumers of vegetables worldwide, China’s vegetable harvest area reached 23.9 million hectares in 2017, which is equivalent to 41% of the total vegetable harvest area in the world (1). The vegetable planting croplands are generally characterized by continuous monocropping and excessive fertilizer management (2). As reported, the annual N-fertilizer input has exceeded around 1200 kg N ha^−1^. However, plant N uptake only accounts for around 20% of input N (3). The fact that there was an adaptive coevolutionary relationship between rhizosphere soil microbes and plant root systems in the long term then formed a reciprocal relationship between plant and soil microbial community (4, 5). However, under continuous monocropping and unreasonable fertilization, the balance of the relationships built by the soil-microbes-plant group is broken, and soil physicochemical and biological characteristics are changed (4, 6–8). In the end, the soil quality of vegetable planting croplands is declining, and the nutrient deficiency is more and more serious, which seriously restricts the sustainable development of vegetable production (4, 9).

Soil monocropping obstacles as typical negative plant-soil feedback that is generally existed in continuous monocropping systems, while soilborne pathogens are largely responsible for the situation (10). In continuous monocropping systems, the accumulation of plant root exudates can provide sufficient specific molecules attracting pathogens over time, leading to their significant colonization and infection in host species (6). Soil-borne pathogens markedly and severely threaten quality and yield in vegetable crops, of which Fusarium oxysporum is the most serious soilborne disease drawing considerable attention for more than a century (11, 12), and it had a broad host range and could adapt to any soil type persisting in the soil for a long time (13). Fusarium oxysporum is commonly referred to as a species complex because it includes many clonally lineages and could cause a series of negative symptoms to the plants, containing vascular wilts, stunting, root rot, yellowing, and even death (6, 14). Based on its devastating impact on crop yield, bacterial genera Fusarium *species*, F. graminearum, and F. oxysporum had been positioned the fourth and fifth among the top 10 economically significant phytopathogenic fungi (15). However, an innovational method for soilborne pathogen suppression (anaerobic soil disinfestation) was introduced effectively to improve soil physical properties and soil pathogens depending on anaerobic soil conditions incorporated with organic matter and antagonism relationships among microorganisms (13, 16–18). Yet, it is a large challenge for protecting vegetable productions from soilborne pathogens in continuous monocropping croplands.

Pyrroloquinoline quinone (PQQ), an organic coenzyme, is generated by a plethora of Gram-negative bacteria and is characterized by high water solubility, thermal stability, stable chemical structure, and high redox potential. It is regarded as a prosthetic group of many redox enzymes in microorganisms, such as methanol dehydrogenase (MDH), glucose dehydrogenase (GDH), fructose dehydrogenase (FDH), sorbitol dehydrogenase (SDH), etc. (19, 20). The present research on PQQ mainly focused on its metabolic processes (21, 22), regulation of ROS (reactive oxygen species) system as a redox factor (23–25). Yet, a very small part turning to soil nutrients modulation or pathogen confrontation *in vitro* (26–28). Still, the improvement effect of PQQ amendment on soil nutrients and soil pathogens, as well as on soil microbial community structure in deficient monocropping soil, remains poorly understood. For instance, it has been reported that partial soil bacteria can dissolve soil inorganic phosphates through the holoenzyme action of GDH-PQQ as a coenzyme of glucose dehydrogenase (28), and some showed a significant effect in depressing crop pathogens based on the PQQ-dependent metabolic process *in vitro* and *in vivo* (26, 29), which suggests that PQQ amendment could improve soil nutrient and to suppress soilborne pathogens of monocropping soils. Nevertheless, the attention to the PQQ effect on soilborne pathogens in monocropping soils has been less concerned, and the underlying microbial mechanism remains unclear, which impedes the development of elimination of soilborne pathogens by adopting a novel external bio-additive.

Given the notable phosphate-solubilization ability and the effective antagonism of the PQQ-production strain *in vitro* (26–28), as well as the significant improvement of soil nutrients (physiochemical characteristic) and soil pathogens within soil flooding conditions by artificial control (16–18). However, the practice of PQQ addition within or without soil aerobic conditions was not carried out in soils, and whether they could yield a synergetic effect on soil nutrients or soil microbial community is still unknown. Thus, PQQ was employed in soils as an organic exogenous compound, and a pot incubation experiment was conducted to examine soil major nutrients and soilborne pathogens from monocropping soils of pepper following PQQ amendment under aerobic and anaerobic conditions. We hypothesized that FL_PQQ (soil irrigated to 100% water holding capacity) anaerobic treatment relative to PQQ aerobic treatment would contribute more to balancing soil nutrients and suppressing soilborne pathogens. We also assumed that inhibitory effects of PQQ amendment on soilborne pathogens could be interpreted by linking to both soil physicochemical properties and microbial community. The main objective of this study is to explore the effects of PQQ amendment under various soil aeration conditions on soil nutrients and soilborne pathogens monocropping pepper soil. This study would help to deepen our understanding of PQQ remediating monocropping obstacle soils by associating with microbial community, which facilitates prospective sustainable agriculture development.

## RESULTS

### Soil physicochemical properties.

An analysis of variance (ANOVA) showed that, relative to control, aerobic and anaerobic PQQ treatments dramatically increased soil pH by 0.74 and 1.13 units, respectively. In terms of soil nutrition, compared to control, the content of available nitrogen (AN) was remarkably enhanced by FL_PQQ treatment, with an increment of 41.81%, while PQQ treatment did not significantly influence it (Table 1, *P* < 0.05). On the contrary, relative to control, PQQ treatment significantly increased the content of available phosphorus (AP) by 144.37%, and FL-PQQ did not significantly affect it. The available potassium (AK) content of PQQ-treated soil decreased by 29.98% compared to control, reaching a statistically significant difference at *P* < 0.05.

**TABLE 1:** Soil physiochemical properties under different treatments

Treatments	AN^*a*^ (mg/kg)	AP (mg/kg)	AK (mg/kg)	pH
Control	165.28 ± 1.98b	133.35 ± 2.36b	550.23 ± 27.90a	5.10 ± 0.06c
PQQ	139.42 ± 2.99c	325.87 ± 82.78a	385.26 ± 21.98b	5.84 ± 0.08b
FL_PQQ	234.39 ± 3.54a	115.35 ± 3.70b	523.78 ± 4.28a	6.23 ± 0.04a

a AN, available nitrogen; AP, available phosphorus; AK, available potassium. Values followed by the same letters are not significantly different according to the Turkey HSD test (*P* < 0.05). Numbers followed by ± are standard errors.

### Abundances of Fusarium oxysporum and pathotrophic fungi.

**FIG 1:**
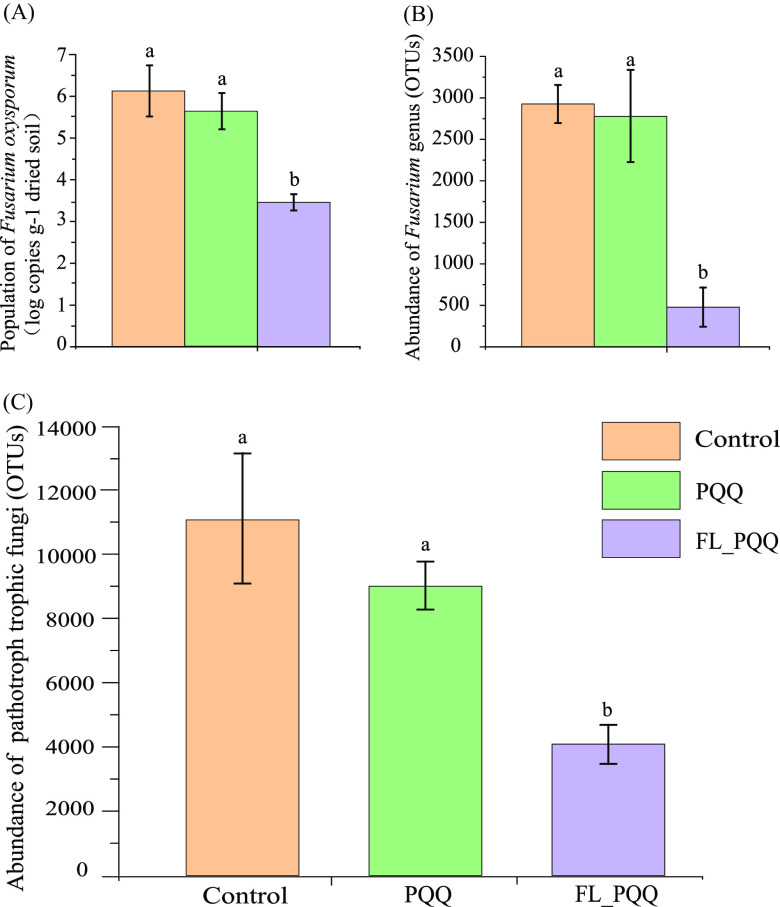
(A) The population size of Fusarium oxysporum and (B) the relative abundance of the genus Fusarium. (C) The relative abundance at OTUs level of the total soil saprophytic-pathotroph fungal community among the three soils, which mainly included five trophic modes, i.e., pathotroph, pathotroph-saprotroph, pathotroph-symbiotroph, pathogen-saprotroph-symbiotroph, pathotroph-saprotroph-symbiotroph annotated by Fungi Functional Guild database (FUNGuild). Vertical bars represent the standard error of the mean.

The population of Fusarium oxysporum in FL_PQQ treatment remarkably decreased by 43.5% compared to control (*P* < 0.05), while there were no statistically significant differences between control and PQQ treatments (*P* > 0.05, Fig. 1A). Similarly, from the aspect of genus level, the abundance of the Fusarium genus was clustered into 2972 OTUs in control, which was not significantly different from that of PQQ treatment (*P* > 0.05) but was significantly higher than that of FL_PQQ treatment (487 OTUs) (*P* < 0.05, Fig. 1B). Moreover, according to trophic modes classification of fungal community, the community with pathotroph mode was found in all treatments, with a range of 4024 to 11277 of OTUs value (Fig. 1C, Table S2). Across both PQQ-added treatments, relative to control, the total pathotrophic fungi size was decreased on average by 41.3%, of which FL-PQQ treatment reached a significant level (*P* < 0.05). Although no significant differences were observed in the abundances of Fusarium oxysporum, fungal Fusarium genera, and pathotrophic fungal communities between control and PQQ treatments, the abundances of the three fungal groups in PQQ treatment were still below those of control, with reductions of 0.5 log copies, 78 and 2061 OTUs abundances, respectively.

### Diversity and richness of soil microbial community.

**FIG 2:**
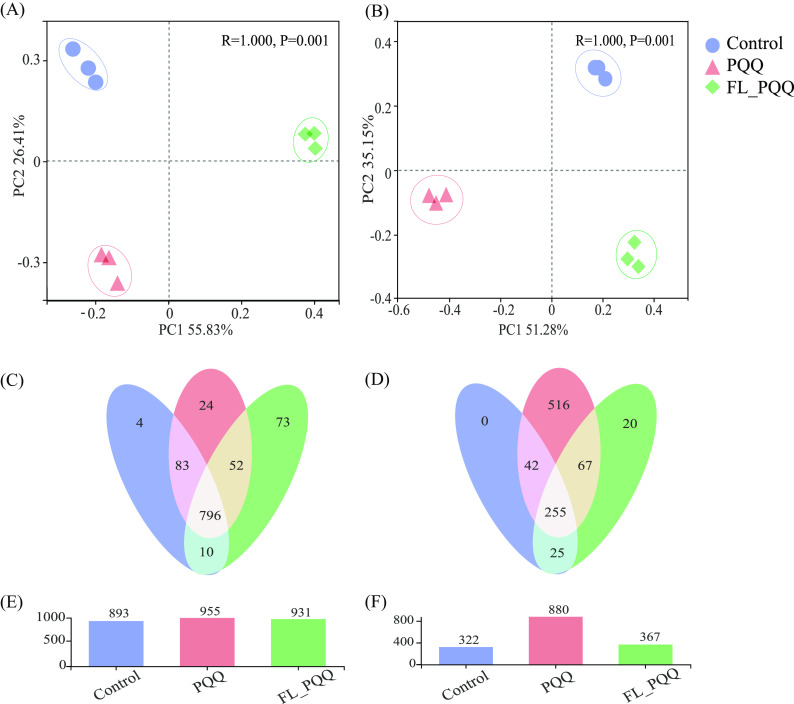
Principal coordinates analysis (PCoA) was for the dissimilarity of the microbial communities ([A] bacteria; [B] fungi) in untreated or treated soils. The percentage of variation given on each axis referred to the explained fraction of total variation. Venn diagram ([C] bacteria; [D] fungi) depicted were unique or shared OTUs among three soils. The histogram showed the total counts of OTUs of (E) bacteria and (F) fungi in three soils.

According to the analysis of variance results for the Shannon diversity index and the Chao and Ace richness estimators, as shown in Table 2, bacterial diversity and richness in PQQ treatment were significantly higher than those of control and FL_PQQ treatments (*P* < 0.05). In addition, relative to control, FL_PQQ treatment remarkably decreased bacterial diversity by 4.28% (*P* < 0.05), while there were no significant differences in bacterial richness indexes (*P* > 0.05). In the aspect of the fungal community, there were significant differences in fungal richness and diversity among different treatments. Concretely, both PQQ-added treatments significantly increased fungal diversity and richness compared to control. However, PQQ treatment had a higher enhancement extent than FL_PQQ treatment. In addition, the number of OTUs for both bacteria and fungi was increased compared to control in PQQ-added treatments (Fig. 2C and D). It was worth noting that the PQQ treatment increased 516 distinctive operational taxonomic units (OTUs) of fungi. The shared OTUs of three treatments (i.e., control, PQQ, FL_PQQ) in the bacterial community and fungal community accounted for 76.39% and 27.57%, respectively (Fig. 2C and D).

**TABLE 2:** The diversity evaluation of microbial community investigated by MiSeq sequencing under different treatments

Treatments	Bacteria			Fungi		
	Shannon^*a*^	Ace	Chao	Shannon	Ace	Chao
Control	5.38 ± 0.12b	845.48 ± 21.34b	865.02 ± 11.29b	3.17 ± 0.13c	256.62 ± 6.84c	261.67 ± 8.77c
PQQ	6.01 ± 0.04a	926.82 ± 3.94a	934.97 ± 7.77a	5.28 ± 0.65a	852.38 ± 32.08a	864.28 ± 38.05a
FL_PQQ	5.15 ± 0.06b	873.92 ± 11.77b	881.47 ± 5.80a	3.96 ± 0.16b	299.18 ± 4.01b	304.23 ± 3.55b

a Values followed by different letters are significantly different according to the Turkey HSD test (*P* < 0.05). Numbers followed by ± standard errors (SE). Shannon indicates the Shannon-Weiner index; ACE represents the abundance-based coverage estimator; Chao shows Chao's species richness estimator.

### Bacterial community composition.

**FIG 3:**
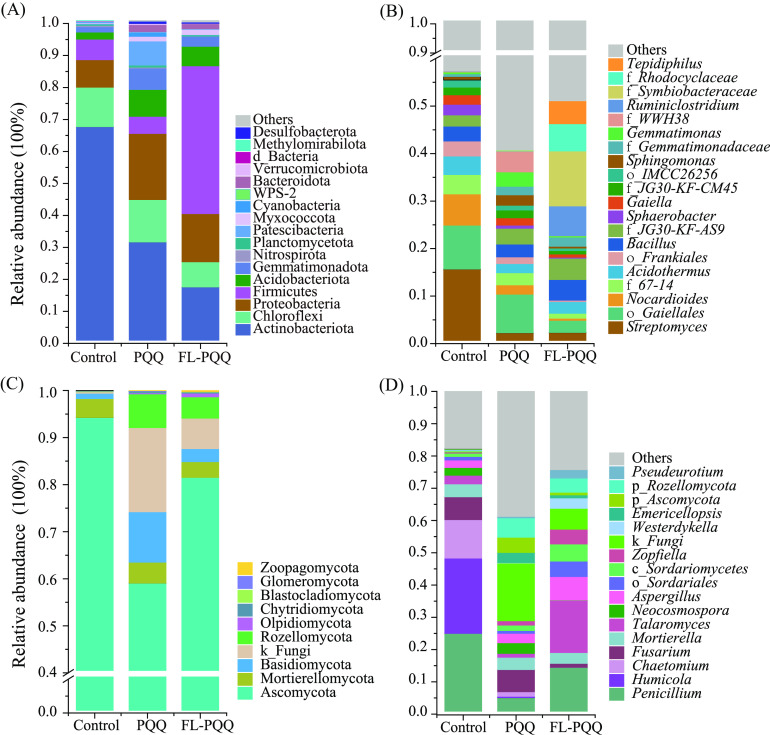
The relative abundance of (A) bacterial phyla and (B) genera, and (C) fungal phyla and (D) genera across three soils. The relative abundance lower than 1% was copolymerized in “Others”. The bacterial genera present in the chart (relative abundance of >1%) both belonged to the Actinobacteria phylum. The fungal genera in the chart mostly belonged to the Ascomycota phylum. The letters “p_,” “c_,” “f_,” and “o_” meant unclear classification at the “phylum,” “class,” “family,” and “order” levels, respectively.

A total of 470,558 high-quality bacterial 16S rRNA gene sequences were detected and clustered into 1,043 OTUs at 97% sequence similarity. Then, 1043 OTUs amounting to 20,951 among soils were retained after further modification for the following analyses. As shown in Fig. 3A, PQQ regimes significantly affected the relative abundances of bacterial phyla, especially Firmicutes and Actinobacteria. In detail, PQQ-related treatments dramatically decreased the relative abundance of phylum Actinobacteria compared to control, with reductions of 54% and 75% for PQQ and FL_PQQ treatments, respectively. On the contrary, the relative abundance of phylum Chloroflexi in all treatments appeared a slight variation of the contribution for total phylum bacteria. The relative abundance of the phylum Firmicutes in FL_PQQ was 46.23% and significantly higher than that of the other treatments (*P* < 0.05). Additionally, relative to control, the relative abundances of the phyla Proteobacteria and Acidobacteria increased by PQQ-added treatments. Noticeably, in all treatments, a certain amount of the phyla Cyanobacteria was only found in PQQ treatment.

At the genus level of the bacterial community (relative abundance > 1%, Fig. 3B), large decreases in the genera of *Streptomyces* (−13.35%) and o_*Gaiellales* (−6.69%) were observed under FL_PQQ treatment compared to control. Therefore, some bacterial genera were enriched alternatively in FL_PQQ, such as *Tepidiphilus* (+4.83%), f_*Rhodocyclaceae* (+5.74%), and *Ruminiclostridium* (+6.25%) (Fig. 3B). Except for the three increased genera mentioned above in FL_PQQ treatment, there were still some other bacterial genera that (relative abundance <1%) showed an increasing trend, i.e., *Thermincola*, *Aneurinibacillus*, *Oxobacter*, *Hydrogenispora*, and f_*Christensenellaceae*, with an increasing range of 0.9% to 5.5% (unpublished data).

Principal coordinate analysis (PCoA) of the OTU levels revealed significant variations in the bacterial community composition among the different treatments (Fig. 2A). Permutational multivariate analysis of variance (PERMANOVA) suggested that the bacterial community composition was significantly influenced by soil AN and pH (Table S3). The PCoA biplot revealed that 82.24% of the total variation in the bacterial community composition was explained by the first two axes of the PCoA, of which PC1 and PC2 explained 55.83% and 26.41%, respectively. The bacterial community in the control group was completely separated from those in the PQQ-added treated groups. Moreover, such completed separation was observed between PQQ and FL-PQQ groups.

### Fungal community composition.

A total of 543,364 high-quality fungal ITS gene sequences were detected and clustered into 925 OTUs at 97% sequence similarity. After further modification, 925 OTUs adding up to 41,718 in each sample were retained for the following analyses. The fungal sequences mainly clustered into the Ascomycota phylum, and the highest relative abundance was observed in the control, accounting for 94.21%, followed by Basidiomycota and Rozellomycota. Compared to control, PQQ-added treatments decreased the relative abundance of Ascomycota, of which PQQ treatment decreased more, with a reduction of 37.67%. On the contrary, PQQ treatment increased Basidiomycota, Rozellomycota, and k_Fungi, especially Rollemycota. In contrast, PQQ-added treatments covered more the relative abundance of Rozellomycota than that of control (Fig. 3C).

In terms of the fungal genus (Fig. 3D), The most abundant genera were *Penicillium*, *Humicola*, *Chaetomium*, Fusarium, *Mortierella*, *Talaromyces*, *Neocosmospora*, Aspergillus, *Cosmospora*, *Westerdykella*, *Pseudeurotium*, etc. Relative to control, the relative abundances of the genera Fusarium, *Humicola*, and *Chaetomiaceae* were significantly decreased by FL_PQQ treatment, while *Talaromyces*, *Pseudeurotium*, Aspergillus, and *Zopfiella* were significantly increased by 6.05, 13.17, 1.28, and 1.60-fold, respectively. In addition, compared to control, PQQ treatment greatly enhanced the relative abundances of the genera unidentified at the expense of decreasing those of *Chaetomium*, *Humicola*, and *Penicillium*.

The distinct changes in the fungal community composition were disclosed by PCoA analysis among the different treatments (Fig. 2B). The first axis, PC1, explained 51.28% of the variations, PC2 explained 35.15% of the variations, and the cumulative contribution rate was 86.43%. The PCoA biplot showed that the fungal community was completely separated among the treatments. PERMANOVA analysis revealed that soil major nutrients (N, P, and K) and pH all dominantly affected the fungal community composition (Table S3). 

### Linkages between soil physicochemical, biotic factors, and pathogens.

**FIG 4:**
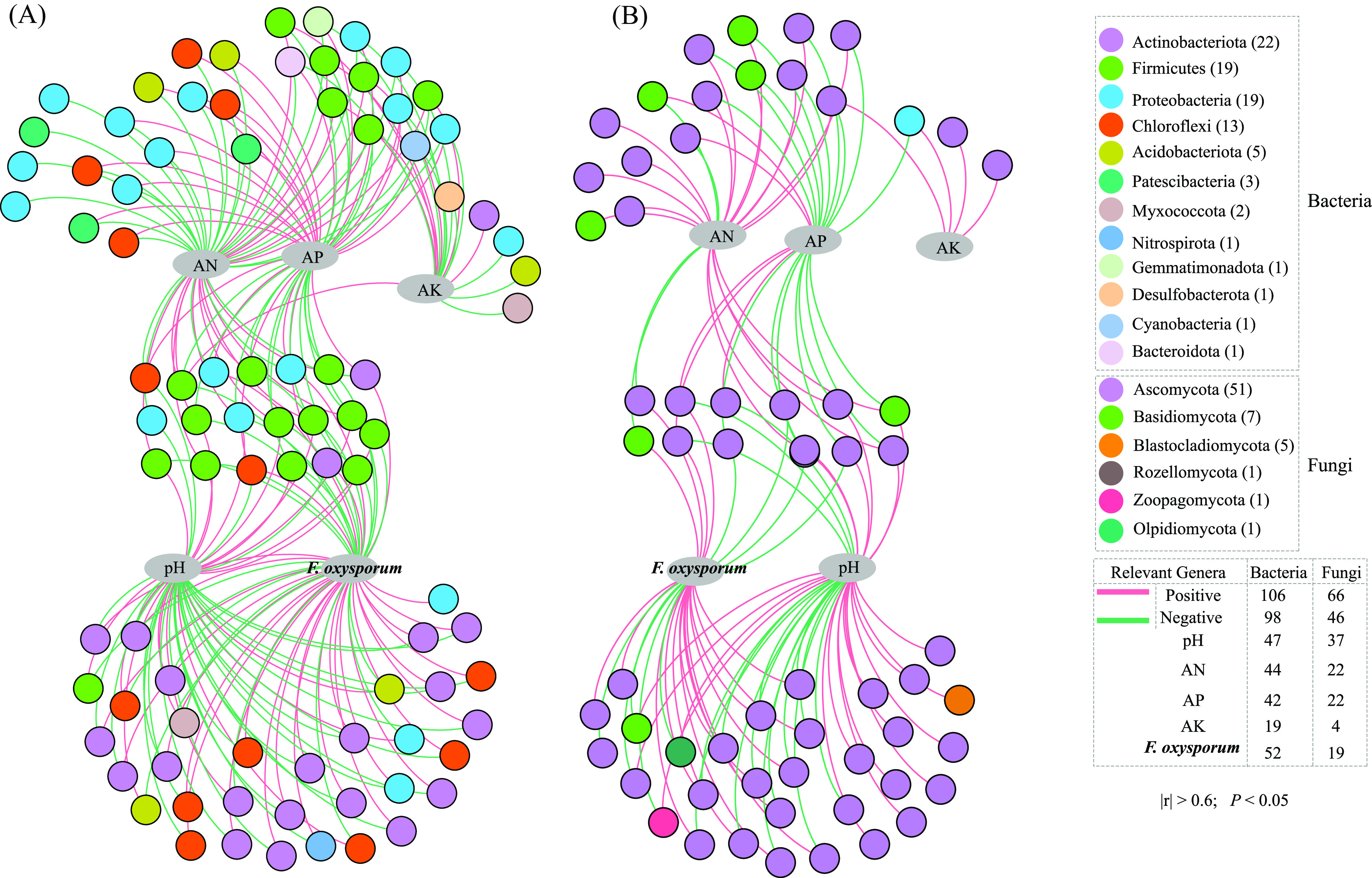
Correlation network of environmental factors and main microbial genera ([A] bacteria; [B] fungi) were based on Pearson correlation analysis (|r| > 0.6, *P* < 0.05). Solid lines signified positive correlation and dotted lines signified negative correlation. Every dot corresponded to a genus of microbes in the network and the dots were colored by phylum.

To clarify relationships between soil environmental factors and microbes at the genus level (top 100 microbial genera), correlation networks with threshold values of |r| > 0.6 and *P* < 0.05 were carried out (Fig. 4). The results showed that AN, AP, and pH played vital roles in reshaping soil microbial community structure, and they significantly related to 83 and 62 genera of bacteria and fungi, respectively. As shown in the correlation network diagram (Fig. 4A), there were 16 genera in the phylum Firmicutes and 17 genera in the phylum Ascomycota which were positively related to AN, while 14 genera belonging to the phylum Proteobacteria were negative (Table S4). AN also had significant correlations with other bacterial genera belonging to the phyla of Actinobacteriota or Chloroflexi which were closely related to soil pH (Table S4). As for AP, it had a significant and positive relationship with 10 genera of the phylum Proteobacteria and others like *Haliangium* and *Gemmatimo nas* genera (Fig. 4A), as well as some fungal genera belonging to the phylum Ascomycota (Fig. 4B, Table S4).

Of note, soil pH, AN, and AP showed a strong connection to F. Oxysporum (Fig. 4). As shown in Fig. 4, a considerable number of genera related to pH were also related to F. Oxysporum, including 34 and 16 genera for bacteria and fungus, respectively, mainly belonging to Actinobacteriota, Chloroflexi, Ascomycota. In addition, there were 18 bacterial genera and 8 fungal genera related to F. Oxysporum which were dependent on AN and AP.

**FIG 5:**
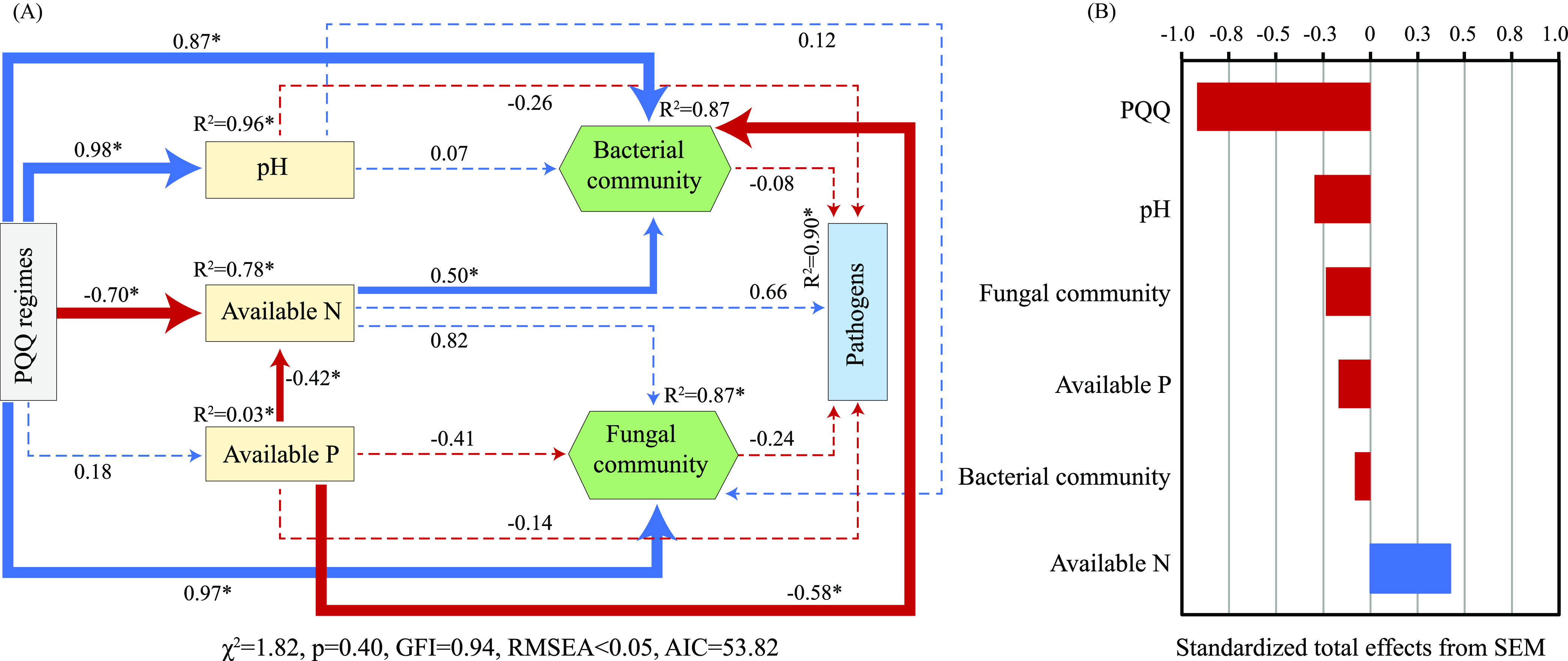
(A) Structural equation model for the effects of soil properties and microbial communities on pathogens from monocropping obstacle soil of pepper under PQQ regimes. Path coefficients (values on the arrows) indicated the relationships between the two variables on both sides of the arrows. Blue and red arrows indicated positive and negative relationships, respectively. Solid and dashed lines represented significant and nonsignificant pathways at a 0.05 significant level, respectively. The width of arrows of solid lines is proportional to the strength of path coefficients. R^2^ values denote the amount of variance explained by the model for the response variables. (B) Standardized total effects (direct plus indirect effects) of variables on pathogens in structural equation model. PQQ regimes represent specific soil conditions of PQQ application, including aerobic and anaerobic conditions.

SEM analysis was employed to reveal how PQQ regimes directly or indirectly affect soil nutrients and microbial communities to influence pathogens from continuous obstacle soils of pepper. The result showed that the SEM explained 90% of the variance for pathogens from the soil, which was well supported by the different goodness of fit metrics used (Fig. 5A). In the model, we found that, although PQQ regimes did not directly affect the pathogens, it was able to influence the size of pathogens indirectly and significantly by regulating soil physicochemical factors and microbial communities (*P* < 0.05, Table S5). The factors significantly affected by PQQ regimes included soil pH, AN, and microbial communities (Fig. 5A). Overall, except for AN having stimulating effects on pathogens, the predictors selected in the model had total negative effects on them, of which the predictor of PQQ had the highest standard total effects, up to −0.92 (Fig. 5B).

## DISCUSSION

### Effects of PQQ regimes on soil microbial community composition.

Generally, the arable soil properties mainly depend on exogenous interference by humans, like fertilization, irrigation, and other agricultural practices, while such disturbances also would influence the formation of soil microbial structure (30, 31). Expectedly, with PQQ application in either aerobic or anaerobic soil conditions, the microbial community structure was significantly altered. Consequently, the anaerobic groups were largely propagated in FL_PQQ treatment, with a relative abundance of 17%, but simply 2.5% and 4.5% for PQQ and control, respectively (Fig. S1). In addition, the aerobic groups in FL_PQQ treatment declined by 20% more than those in control. Thereinto, the major anaerobic microbial groups included *Thermincola*, *Hydrogenispora*, and *Oxobacter*. The result could ascribe to a synergistic effect of anaerobic conditions and PQQ addition. However, this work did not design irrigated anaerobic treatment alone. Thus, we were unable to quantify the effect size of PQQ on microbial communities in irrigated anaerobic conditions and those closely related to anaerobic conditions. As previously reported, the anaerobic condition indeed favored the enrichment of the above three genera. Moreover, their metabolic activity could affect other living processes, especially those that had an antagonized relationship with them (17, 32–34).

Based on the indices of richness and diversity analysis, the PQQ amendment seemed to have an inducing force of increasing bacterial and fungal richness and diversity indices (Shannon, Ace, Chao), especially to fungi. In detail, there were 516 unique OTUs found in the PQQ treatment, accounting for 45% relative abundance, while FL_PQQ and control groups had few or none (Fig. 2). As shown in Fig. 3D, some functionally and significantly different genera of fungi, like saprotrophic *Penicillium* and Aspergillus (35), biocontrol agents of *Zopfiella* and *Talaromyces* (16, 36) accounted for a large portion of both PQQ treatments, which was important for the cycling of our resistance to pathogens in soil (37–39). Besides, Gram-negative bacteria, such as *Gemmatimonas*, f_*Gemmatimonadaceae*, *Sphingomonas*, f_*WWH38*, etc., were dominated in both PQQ-treated soils (+10% relative to control) while PQQ originally exists in Gram-negative microbes, which meant PQQ induced the gram-negatives to be enriched in soils.

In brief, after exogenous addition and environment control to the soil, soil properties and microbial community structure evolved following their interaction effect, and ultimately reached a relative balance between soil environment and soil microbes.

### Effects of PQQ regimes on soil main nutrients.

PQQ could significantly improve soil physicochemical properties when it was applied to the 10-year monocropping soil of peppers (Table 1). After PQQ treatments, an apparent change was an increase in soil pH, especially in FL_PQQ treatment, with an increase of 1.13 units relative to control. This could be due to the proliferation of anaerobic microorganisms. As reported by Huang et al. (16) and Ueki et al. (18), such functional microbes with their metabolic activity could lead to soil microenvironmental change, resulting in soil pH raising. Sole PQQ addition also slightly increased soil pH compared to control, which could be explained by the PQQ-induced increase of microbial groups (21).

Soil pH, as an important physicochemical factor, could affect microbial activities, nutrient availability, soil fertility, and plant growth (40–42). In the study, correlation analysis indicated that many microbial genera were significantly related to pH, AN, and AP (Fig. 4). Among these significant genera, some participated in the cycling of soil nutrients, regulating the contents of soil available nutrients. For the N cycling process in nature, the main way was the nitrification/denitrification process which generally happened in aerobic/anaerobic environments, respectively (42, 43). After FL_PQQ treatment, some denitrifying bacteria (i.e., *Bacillus*, *Tepidiphilus*, *Clostridium_sensu_stricto_1*) were enriched, which meant a strong denitrifying ability, and soil AN existed mostly in form of NH_4_^+^-N. Compared to PQQ treatment, the increase in AN content under FL_PQQ treatment was probably owing to the large denitrifying bacterial community which could transform NO_3_-N into NH_4_^+^-N by denitrification process during the anaerobic condition, resulting in the loss of available nitrogen form (NO_3_-N), but the accumulation of NH_4_^+^-N form. Meanwhile, a high soil pH value in FL_PQQ treatment also proved that NH_4_^+^ was the main source of available nitrogen. Previous studies showed that ammonia oxidation rates were decreased following an increase in soil pH (44, 45). Moreover, other bacteria such as the decomposer of cellulose (*Tepidiphilus* and *Ruminiclostridium*) and the producers of organic acids (*Oxobacter* and *Hydrogenispora*) were significantly positive for AN. Therefore, they could also help improve soil AN during their metabolizing process.

Soil P availability was regulated by both PQQ and the ambient microbial community. PQQ treatment significantly improved soil AP, compared to control, which was in line with the result reported by Jiao et al. (46). The previous study showed that the content of total P of plant and soil AP in maize treated with DH5α (PQQ) increased, mainly because the DH5α (PQQ) could aid some phosphorus solubilizing bacteria (PSB) to secret gluconic acid, this organic acid, making more insoluble P become soluble P. The PQQ compound mainly existed in Gram-negative bacterium, which was dramatically increased after PQQ addition, +39% (PQQ), +30% (FL_PQQ) against control (Fig. 5). Strangely, soil AP increased only in the aerobic condition of PQQ, while in an anaerobic environment, FL_PQQ treatment, the content of AP in soil was almost as much as control, which meant the necessary of aerobic condition for PQQ to dissolve P from insoluble form. The higher AP content was available in aerobic PQQ soil (325.87 mg/kg) rather than in control soil (133.35 mg/kg) or FL_PQQ treated soil (115.35 mg/kg) (Table 1). Meanwhile, it could be found that microbes positively related to AP almost were aerobic, e.g., *Bradyrhizobium* (0.63*), *Sphingomonas* (0.90***), Dyella (0.85**), *Gemmatimonas* (0.82**) of bacterial genera according to previous studies (47–49). These dominant microbes in PQQ-treated soil were important for the improvement of soil P nutrient availability. Furthermore, Liu et al. (50) reported the phylum Cyanobacteria could recruit the PSBs nearby, which played an important ecological role in P mineralization. In this study, the relative abundance of Cyanobacteria considerably increased in PQQ treatment for the addition of PQQ within the aerobic condition (Fig. 3A).

### Effects of PQQ regimes on soil pathotrophic fungi populations.

PQQ treatments were proved effective against soil pathotrophic fungal communities, owing to the PQQ-inducing beneficial microbes and their recruited partners having a dominant position under niche competition circumstances. According to previous studies, the soil microbial community played a vital role in maintaining soil health and suppressing plant diseases (51, 52). Meanwhile, the soil organisms also affected each other in the ways of antagonism, competition, and symbiosis (53). The beneficial microbes for plant growth and health are well documented for the phyla Proteobacteria, Firmicutes, and Acidobacteriota (54, 55), the abundance of which was increased in two PQQ-added treatments against control, especially Firmicutes dominated in anaerobic treatments (FL_PQQ) (Fig. 3A), which was consistent with another report (56). After PQQ application into the soil, accompanied by niche competition of soil resident microorganisms, soil available nutrients (e.g., AN, AP) were improved simultaneously, which was also helpful to forming new microbial community structure, and the finally reshaped microbial community and soil properties proved the significance of the PQQ treatments.

Compared to PQQ, however, FL_PQQ treatment remarkably lowered the OTU relative abundance of the total pathotrophic fungal community. The fungal genus Fusarium and Fusarium oxysporum species fell percentage points of 56, 83, and 39, respectively, and could induce more antagonists belonging to the phyla of Firmicutes and Proteobacteria (57). For instance, *Tepidiphilus*, *Thermincola*, and *Hydrogenispora* play a leading role in the process of the anaerobic fermentation process (57–59). Others, like *Oxobacter*, and *Bacillus* can produce organic acids (60, 61) or various antibiotics (62, 63), which were supposed to contribute to pathogen inactivation (64). Correlation network analysis again proved that the above five bacterial genera were strongly negative to F. Oxysporum, with the correlation value of −0.82**, −0.74*, −0.92***, −0.86**, −0.83**, respectively (*significant at *P* < 0.05; **significant at *P* < 0.01; ***significant at *P* < 0.001) (Fig. 4). Moreover, FL_PQQ treatment propagated dramatically some anaerobic bacteria (6.8-fold quantity compared to control) (Fig. S1). Huang et al. (16) and Ueki et al. (18) reported that the suppression effect on soil pathogens under anaerobic reductive soil conditions was related to an increase of anaerobic bacteria, which meant the anaerobic microbes in soils were important for the disease control.

In addition to the propagation of some beneficial bacteria, some functional fungi thrived after cooperating PQQ amendment with the anaerobic condition, such as *Talaromyces*, *Zopfiella*, and *Coniochaeta* (Fig. 3D), and they were also negatively related to F. Oxysporum species, that is −0.45, −0.42, −0.68* correlation values. As a previous study reported, these three fungal genera showed markedly antifungal activity against Fusarium oxysporum, Rhizoctonia solani, or Pythium aphanidermatum for various crops (36, 65, 66). Further, the genus *Talaromyces* was considered an important biological agent of soilborne fungal diseases and have already been widely used in agriculture (36). Although the abundance of soil pathogenic fungi was no significant differences between control and aerobic PQQ treatment, the latter was lower than the former, which might be ascribed to the induced special microorganisms of Gram-negative bacteria or the unique functional fungi by PQQ amendment that might be helpful to against soilborne pathogens by the allelopathic interactions or competition for the living space. To sum up, SEM analysis consistently reflected that PQQ plays an important role in suppressing soil pathogens by the indirect ways of first regulating soil nutrients to the microbial community, and then further affecting soil pathogen growth.

This study provided an insight into the responses of soil major nutrients and soilborne pathogens under monocropping obstacle soil of pepper to PQQ amendment in both aerobic and anaerobic soil conditions by linking to the microbial community. Consistent with the initial hypothesis, the study found that, relative to the aerobic PQQ treatment, anaerobic PQQ treatment more benefits suppression of soilborne pathogens, and the two PQQ treated patterns (i.e., aerobic or anaerobic pattern) had different effects on soil main nutrients. Based on the second hypothesis, we demonstrated that PQQ application changed the soil microbial community composition by regulating soil property as reflected in SEM analysis, thereby decreasing the abundances of Fusarium oxysporum and pathotrophic fungal community, of which FL_PQQ treatment was more significant, while the weak inhibiting effect of PQQ might cause by recruiting beneficial microbes. Relative to PQQ, the anaerobic environment in FL_PQQ was still considered an important factor responsible for pathogen suppression as reported (18) in which the anaerobic microbes belonging to Firmicutes phylum (bacteria) and Ascomycota phylum (fungi) as antagonists largely thrived and contributed to the suppression of soilborne pathogens. Nevertheless, some more advanced technical approaches such as metabolomics and transcriptional profiling analysis are expected to further explore the mechanism underlying the effects of PQQ amendment on soil soilborne pathogens from agricultural monocropping obstacle soils.

## MATERIALS and METHODS

### Experimental sites.

The soil used in this study was collected from intensive greenhouse fields with a loamy texture, located at the experiment site of Hunan Academy of Agricultural Sciences, Changsha, Hunan, China (113°01’ E, 28°12’ N), where excessive fertilization and continuous pepper monocropping in the past 10 years resulted in the frequent occurrence of damping-off disease. The basic soil properties collected from the topsoil of the site (0 to 20 cm) were pH 5.7, organic matter (OM) 33.28 g/kg, total nitrogen (TN) 1.84 g/kg, total phosphorus (TP) 2.26 g/kg, total potassium (TK) 14.28 g/kg.

### Experimental design.

The pot incubation experiment was conducted from August to September 2019 and was comprised of three treatments with three replicates, generating a total of 9 treatment plots. The three treatments are as followed: (i) control, untreated continuous obstacle soil with water to soil ratio of 15% to 22% (wt/wt); (ii) PQQ, soil treated with 200 mL 2000 nmol/L PQQ without irrigation (but same water holding rate with control) or plastic film covering; (iii) FL_PQQ, soil irrigated to 100% water holding capacity, and incorporated with 200 mL 2000 nmol/L PQQ and vacuum sealed with plastic film.

All treatments were conducted in square PVC pots (25 × 25 × 10 cm) containing 4 kg of field soil with an initial moisture content of 12.5% (v/w). After the incubation procedure of soil at 30°C in the greenhouse for 20 days, soil samples were destructively collected from the triplicate soil microcosm of each treatment and then dried, finely grounded, and homogenized. Each soil sample was divided into two parts. One portion was stored at 4°C for physicochemical properties, and the remaining sample was stored at −80°C for sequencing analysis.

### Soil physicochemical properties.

Soil pH was measured with soil to water ratio of 1:2.5 (wt/vol) using an S220K pH meter (Mettler-Toledo International Inc., China) (50). Soil available phosphorus (AP) and available potassium (AK) were tested as the details by Fan et al. (67) by instruments of a UV spectrophotometer (Unico Instrument Co., LTD, WFZUV-4802H, Shanghai, China) and a flame photometer (Sherwood M410, England), respectively. Soil available nitrogen (AN) was determined by semimicro Kjeldahl digestion (16). Soil organic matter (OM) was digested by H_2_SO_4_-K2Cr2O7 and quantified by titrimetry with FeSO_4_ as a reductive agent (68, 69).

### DNA extraction and MiSeq sequencing.

Following the manufacturer’s instruction of the E.Z.N.A. soil DNA kit (Omega Bio-Tek, USA), the extraction of total DNA in soil was executed. The extracted DNA was checked on 1% agarose gel, and DNA concentration and purity were determined with NanoDrop 2000 UV-vis spectrophotometer (Thermo Scientific, USA). All DNA samples were stored at −20°C for further microbiological analyses. The V3 to V4 hypervariable regions of bacterial 16S rRNA gene were amplified using primer pair of 338F and 806R (70), and the fungal ITS1 regions were targeted using the primers of ITS1F and ITS2R (Table S1) (71). The reaction mixture and thermal profile of PCR amplifications were performed by an ABI GeneAmp 9700 PCR thermocycler (ABI, USA) refereeing to the published paper described by Xu et al. (71). PCRs were performed in triplicate. After successful amplification, the PCR products were extracted from 2% agarose gel and purified using the AxyPrep DNA Gel Extraction kit (Axygen Biosciences, USA) according to protocol and quantified using Quantus Fluorometer (Promega, USA). Purified amplicons were pooled in equimolar, and paired-end sequenced (2 × 300) on an Illumina MiSeq platform (Illumina, USA) as the standard protocols by Majorbio Bio-Pharm Technology Co. Ltd. (Shanghai, China). The raw sequencing reads were further processed with Trimmomatic for reading trimming and filtering, and with FLASH for reading merging, as the following criteria. The 300-bp reads were truncated at any site based on an average quality score of <20 and a >50 bp sliding window, and the truncated reads shorter than 50 bp or the reads containing ambiguous characters were discarded. Only overlapping sequences longer than 10 bp were assembled according to their overlapped sequence. Samples were distinguished according to the barcode and primers, and the sequence direction was adjusted for the exact barcode matching. The resulting sequences were clustered into operational taxonomic units (OTUs) with a 97% similarity cutoff using UPARSE (version 7.1, http://drive5.com/uparse/), and chimeric sequences were identified and removed. OUTs representative sequences were taxonomically annotated through RDP Classifier (version 2.2 http://sourceforge.net/projects/rdp-classifier/) against the 16S rRNA database with a 0.7 confidence threshold. The fungal ITS sequences were analyzed using Unite (Release 7.2 http://unite.ut.ee/index.php). As indicated above the 16S DNA processing, the quality filtering, similarity clustering, and chimera removal of the ITS region were finished, and then we obtained the taxonomic information of representative OTUs (from the kingdom, phylum, class, order, family genus to species level). The raw OTU table was carried out standardized procedure based on the minimum OTU sum among soil samples.

### Real-time quantification PCR assay for Fusarium oxysporum.

Quantitative PCR amplification was performed on an ABI7500 real-time PCR system (Applied Biosystems, USA) (16). In detail, the 20 μL reaction mixture contained 16.5 μL of ChamQ SYBR Color qPCR Master Mix (2×) (Vazyme Biotech Co., Ltd., China), 0.8 μL of each primer (5 μM), and 2 μL of template DNA. The primer pair of ITS1F/AFR308R (Table S1) was used to target the ITS region for Fusarium oxysporum (65). Serial dilutions of the plasmid DNA were used to generate standard curves with a slope value of −3.4933. The abundance value of Fusarium oxysporum was subjected to log_10_-transformed and expressed by log copies per gram of dried soil.

### Bioinformatic processing and statistical analyses.

For microbial alpha diversity analysis, the indexes (ACE, Chao, and Shannon) were all calculated by Mothur software (version 1.30.1). A multiple range test of The Turkey HSD test was used to examine the differences in soil physicochemical parameters, Fusarium oxysporum abundances, and microbial community diversity among the treatments using SPSS (version 26.0, USA, 2012). Venn diagrams of the shared and specific OTUs counts among treatments were generated by the online website http://bioinformatics.psb.ugent.be/webtools/Venn/. Principal coordinate analysis (PCoA) was conducted at OTU levels using Bray-Curtis distance with the R package of vegan and visualized the dissimilarities in the bacterial and fungal community structures among soil samples. To estimate the correlation coefficients between microbial compositions and environmental factors, the ‘corr.test’ function of the R package (psych) was used (method with “Pearson,” adjust with “fdr”). Correlation network analyses of microbes and environmental factors were visualized by Cytoscape 3.7.2 based on Pearson correlation analysis (|r| > 0.6, *P* < 0.05). Permutational multivariate analysis of variance (PERMANOVA) was carried out by SPSS 26. at OTU levels to identify the main environmental factor influencing microbial community structure. The structural equation modeling (SEM) was used to evaluate the direct and indirect relationships between soil environmental factors, microbial community, and pathogens from the soils of PQQ regimes based on the generalized least-squares method via AMOS 20.0 (AMOS IBM, USA). The overall goodness-of-fit of model fits was assessed by a series of parameters, including nonsignificant χ^2^ test (*P* < 0.05), root mean square error of approximation (RMSEA, *P* > 0.05), goodness-of-fit index (GFI > 0.9), and Akaike information criteria (72).

### Functional annotation of the microbial community.

We further analyzed the functional structures of bacterial and fungal communities in all treatments, the functional traits for bacterial and fungal groups were obtained from the online websites of Bugbase (https://bugbase.cs.umn.edu/index.html) and FUNGuild (https://cloud.majorbio.com/), respectively (73, 74). Specifically, the bacterial community was kept in six classes, namely, Gram-positive, Gram-negative, oxidative stress-tolerant and aerobic, anaerobic, facultatively anaerobic groups. For the fungal community, we kept 475 OTUs with a confidence ranking of “highly probable” and “probable,” accounting for 51.35% of total fungi OTUs, to reach high accuracy. We only focused on the functional guilds related to soil quality and plant growth health, including pathotroph (55 OTUs), pathotroph-saprotroph (22 OTUs), and pathotroph-symbiotroph (7 OTUs) pathogen-saprotroph-symbiotroph (7 OTUs) and pathotroph-saprotroph-symbiotroph (74 OTUs). Treatment effects on the relative abundance of key microbial functional groups were presented in Fig. 1 and Table S2.

### Data availability.

Sequence data for the amplicon libraries from this study have been deposited in the NCBI Sequence Read Archive (SRA) database with accession number PRJNA665924.
