# Supplementary material for: Insights into Pyrroloquinoline Quinone (PQQ) Effects on Soil Nutrients and Pathogens from Pepper Monocropping Soil under Anaerobic and Aerobic Conditions
Source: Microbiol Spectr. 2022 Jul 19;10(4):e00933-22. doi: 10.1128/spectrum.00933-22 (PMC9430733; doi:10.1128/spectrum.00933-22)
Supplement: Supplemental file 1 — Supplemental material. Download spectrum.00933-22-s0001.pdf, PDF file, 0.3 MB [file spectrum.00933-22-s0001.pdf]

Insights into pyrroloquinoline quinone (PQQ) effects on soil nutrients and pathogens from pepper monocropping soil under anaerobic and aerobic conditions

Xin Li[a], Mingxing Zhang[c] Qingzhuang Zhang[c], Fangjun Tan[c], Zheng Gong[c], Yunhe Xie[b,d,e], Yu Tao[a], Jie Chen[a, b]

[a] Hunan Academy of Agricultural Sciences, Changsha 410125, Hunan, China

[b] Hunan Institute of Agricultural Environment and Ecology, Changsha 410125, Hunan, China

[c] Hunan Vegetables Research Institute, Changsha 410125, Hunan, China

[d]Key Laboratory for Agro-Environment in Midstream of Yangtze Plain, Ministry of Agriculture, Changsha, P.R.China

[e]Hunan Key Laboratory of Agro-Farmland Heavy Metal Pollution Control and Remediation, Hunan, China.

*** Corresponding Author (Jie Chen & Yu Tao)**

Tel.: +86 0731 84692900, fox: +86 0731 84691725, E-mail: royalmanjie@gmail.com

Tel.: +86-0731 84691212, fox: +86-0731 84691212, E-mail: ty123@webmail.hzau.edu.cn

To be submitted **to *Microbiology Spectrum***

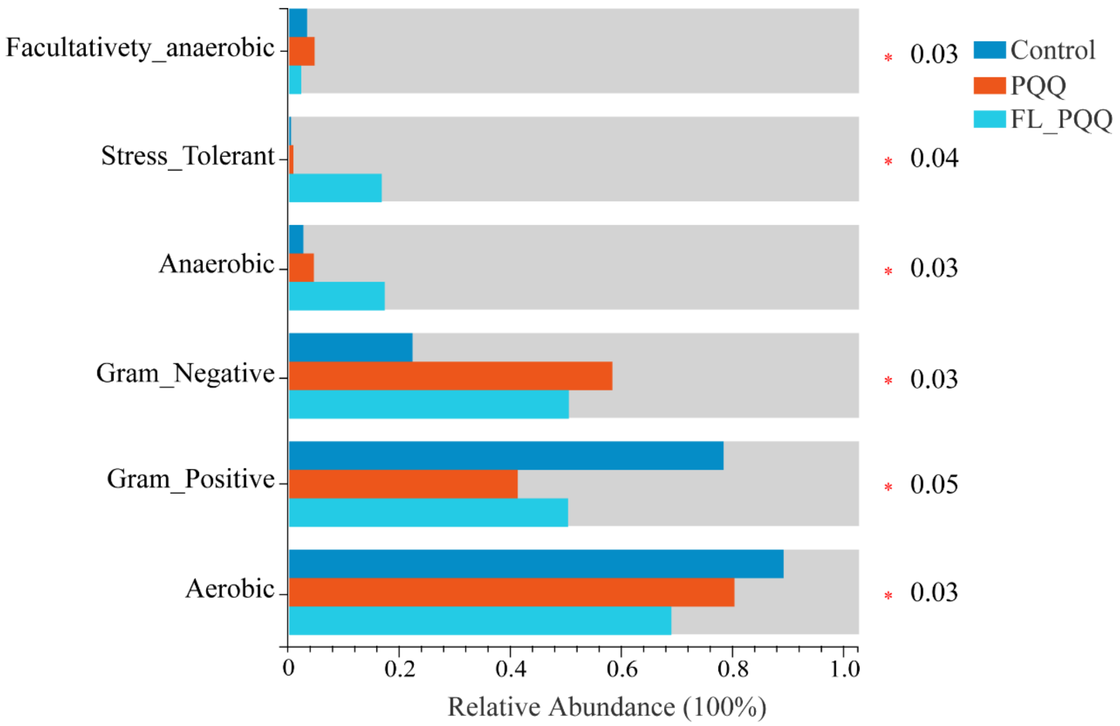

Fig. S1. The relative abundance of the predicted bacterial phenotypes across three soils based on BugBase database.

Table S1. Primers used for analysis of amplifier sequencing in this study.

Targets	Primers	Sequence (5'-3')
16S rRNA	338F	ACTCCTACGGGAGGCAGCAG
	806R	GGACTACHVGGGTWTCTAAT
ITS1	ITS1F	CTTGGTCATTTAGAGGAAGTAA
	ITS2R	GCTGCGTTCTTCATCGATGC
Fusarium oxysporum	ITS1F	CTTGGTCATTTAGAGGAAGTAA
	AFR308R	CGAATTAACGCGAGTCCCAAC

Table S2. Pathotrophic fungal groups and their relative abundance across three soil samples annotated by Fungi Functional Guild database (FUNGuild).

Trophic Mode	Guild	CK	PQQ	FL_PQQ
Pathogen-Saprotroph-Symbiotroph	Animal Pathogen-Endophyte-Fungal Parasite-Plant Pathogen-Wood Saprotroph	52	236	7
Pathotroph	Animal Parasite-Fungal Parasite	27	91	321
	Animal Pathogen	29	296	59
	Fungal Parasite	330	148	693
	Plant Pathogen	1016	1105	214
Pathotroph-Saprotroph	Animal Endosymbiont-Animal Pathogen-Endophyte-Plant Pathogen-Undefined Saprotroph	2	13	2
	Animal Pathogen-Plant Pathogen-Soil Saprotroph-Undefined Saprotroph	1	349	0
	Animal Pathogen-Plant Pathogen-Undefined Saprotroph	18	276	39
	Animal Pathogen-Undefined Saprotroph	4	91	77
	Dung Saprotroph-Plant Parasite-Soil Saprotroph-Undefined Saprotroph-Wood Saprotroph	20	6	86
	Endophyte-Lichen Parasite-Plant Pathogen-Undefined Saprotroph	0	14	0
	Fungal Parasite-Plant Pathogen-Plant Saprotroph	1	167	0
	Fungal Parasite-Wood Saprotroph	887	126	0
	Plant Pathogen-Plant Saprotroph	0	8	0
	Plant Pathogen-Undefined Saprotroph	8	5	0
	Plant Pathogen-Wood Saprotroph	0	7	0
Pathotroph-Saprotroph-Symbiotroph	Animal Pathogen-Dung Saprotroph-Endophyte-Epiphyte-Plant Saprotroph-Wood Saprotroph	5220	677	1521
	Animal Pathogen-Dung Saprotroph-Endophyte-Lichen Parasite-Plant Pathogen-Undefined Saprotroph	2	35	43
	Animal Pathogen-Dung Saprotroph-Endophyte-Plant Saprotroph-Soil Saprotroph-Wood Saprotroph	33	16	0
	Animal Pathogen-Endophyte-Ericoid Mycorrhizal-Plant Pathogen-Wood Saprotroph	0	8	0
	Animal Pathogen-Endophyte-Fungal Parasite-Plant Pathogen=Wood Saprotroph	49	16	80
	Animal Pathogen-Endophyte-Lichen Parasite-Plant Pathogen-Soil Saprotroph-Wood	2973	2895	488

	Saprotroph			
	Animal Pathogen-Endophyte-Lichen Parasite-Plant Pathogen-Wood Saprotroph	111	576	166
	Animal Pathogen-Endophyte-Plant Pathogen-Undefined Saprotroph	71	3	86
	Animal Pathogen-Endophyte-Plant Pathogen-Wood Saprotroph	16	536	10
	Animal Pathogen-Soil Saprotroph	0	50	0
	Ectomycorrhizal-Fungal Parasite-Soil Saprotroph-Undefined Saprotroph	0	0	10
	Endomycorrhizal-Plant Pathogen-Undefined Saprotroph	127	260	0
	Endophyte-Dung Saprotroph-Lichen Parasite-Litter Saprotroph-Plant Pathogen-Soil Saprotroph-Wood Saprotroph	0	78	4
	Endophyte-Lichen Parasite-Undefined Saprotroph	0	62	0
	Endophyte-Plant Pathogen-Undefined Saprotroph	4	6	2
	Endophyte-Plant Pathogen-Wood Saprotroph	8	9	0
	Fungal Parasite-Undefined Saprotroph	219	861	92
Pathotroph-Symbiotroph	Animal Pathogen-Clavicipitaceous Endophyte-Fungal Parasite	0	6	0
	Endophyte-Plant Pathogen	50	35	25
	Ericoid Mycorrhizal	0	150	0
	Total	11277	9216	4024

Table S3. Differences effects of the environmental factors on microbial community structure across three treatments at OTU levels.

Soil properties	Bacteria					Fungi			
	Mean Sqs	F.Model	R^2	P.value		Mean Sqs	F.Model	R^2	P.value
AN	0.681	7.871	0.529	**0.013**	AP	0.726	3.787	0.351	**0.023**
pH	0.585	5.848	0.455	**0.017**	AK	0.892	5.312	0.431	**0.023**
AP	0.290	2.035	0.225	0.088	AN	0.957	6.036	0.463	**0.023**
AK	0.305	2.171	0.236	0.118	pH	0.709	3.654	0.343	**0.035**

Note: AN, available nitrogen; AP, available phosphorus; AK, available potassium.

Table S4. List of phylum and genus of bacteria and fungi in correlation network based on *Pearson* correlation analysis at |r| > 0.6, *P* < 0.05.

		Bacteria	Fungi
AN	P	**Firmicutes**: f_Symbiobacteraceae, Bacillus, Ruminiclostridium, Thermincola, f_Hungateiclostridiaceae, Hydrogenispora, f_Christensenellaceae, Clostridium_sensu_stricto_1, Oxobacter, f_Limnochordaceae, Romboutsia, Clostridium_sensu_stricto_12, o_Desulfotomaculales;<br>**Proteobacteria**: f_Rhodocyclaceae, Tepidiphilus;<br>**Chloroflexi**: f_JG30-KF-AS9, Ktedonobacter;<br>**Actinobacteriota**: Luedemannella;<br>**Acidobacteriota**: Geothrix, o_AKIW659;	**Ascomycota**: Talaromyces, Aspergillus, o_Sordariales, Westerdykella, Pseudeurotium, Sagenomella, Cosmospora, Melanocarpus, f_Sordariaceae, Mycothermus, Ramophialophora, o_Eurotiales, Scedosporium, Arthrographis, Cercophora, Gymnascella, f_Gymnoascaceae;<br>**Basidiomycota**: o_Agaricales, c_Agaricomycetes, f_Strophariaceae;<br>**Olpidiomycota**: Olpidium;
	N	**Proteobacteria**: Sphingomonas, o_Elsterales, f_SC-I-84, Ellin6067, Dyella, Bradyrhizobium, f_Xanthobacteraceae, Reyranella, Pseudolabrys, MND1;<br>**Chloroflexi**: f_JG30-KF-CM45, c_KD4-96, c_Gitt-GS-136, c_JG30-KF-CM66, f_Roseiflexaceae, o_C0119, c_TK10, c_OLB14;<br>**Actinobacteriota**: o_Gaiellales, f_67-14, Gaiella, o_IMCC26256, c_Acidimicrobiia, Mycobacterium, Solirubrobacter, Knoellia, f_Geodermatophilaceae;<br>**Acidobacteriota**: o_Vicinamibacterales, Bryobacter;<br>**Patescibacteria**: f_WWH38;<br>**Nitrospirota**: Nitrospira;<br>**Gemmatimonadota**: Gemmatimonas;	**Ascomycota**: Fusarium, Neocosmospora, Gibberella, Fusicolla, Alternaria, f_Nectriaceae, Trichoderma, Acremonium, Trichocladium, Scutellinia, Schizothecium, Thelonectria, Acrocalymma, Metarhizium, f_Chaetomiaceae;<br>**Basidiomycota**: Lentinula, Saitozyma, f_Mycenaceae;
AP	P	**Proteobacteria**: Sphingomonas, f_SC-I-84, Ellin6067, Bradyrhizobium, f_Xanthobacteraceae, f_Xanthobacteraceae, f_Comamonadaceae, Pseudolabrys, MND1, f_Micropepsaceae, Phenylobacterium;<br>**Patescibacteria**: f_WWH38;<br>**Myxococcota**: Haliangium;<br>**Gemmatimonadota**: Gemmatimonas, f_Gemmatimonadaceae;<br>**Desulfobacterota**: Citrifermentans;<br>**Cyanobacteria**: Leptolyngbya_EcFYyyy-00;<br>**Chloroflexi**: c_KD4-96, f_Roseiflexaceae, o_C0119;<br>**Bacteroidota**: Flavihumibacter;<br>**Acidobacteriota**: Bryobacter, f_Acidobacteriaceae_Subgroup_1;	**Ascomycota**: Neocosmospora, Fusicolla, Alternaria, f_Nectriaceae, Trichoderma, Knufia, Acremonium, Pyrenochaeta, Pichia, Gymnoascus, o_Pleosporales, Trichocladium, Cephalotrichum, Didymella, Fusidium, c_Archaeorhizomycetes, Scutellinia, Schizothecium, o_Xylariales, Oidiodendron, Setophoma, Thelonectria, Epicoccum, Metarhizium, Stagonosporopsis;<br>**Basidiomycota**: Lentinula, Saitozyma, f_Mycenaceae, Clitopilus, Cutaneotrichosporon, f_Thelephoraceae, f_Ceratobasidiaceae, Vishniacozyma, Apiotrichum;<br>**Rozellomycota**: o_Branch02;

	N	**Firmicutes**: Romboutsia;	/
AK	P	**Firmicutes**: Romboutsia, Tumebacillus;<br>**Actinobacteriota**: Actinoallomurus;	**Ascomycota**: Penicillium;
	N	**Proteobacteria**: Sphingomonas, f_SC-I-84, Ellin6067, Dyella, Bradyrhizobium, f_Xanthobacteraceae, f_Comamonadaceae, Pseudolabrys, MND1, f_Caulobacteraceae, f_Micropepsaceae, Phenylobacterium;<br>**Patescibacteria**: f_WWH38, o_Saccharimonadales, f_LWQ8;<br>**Myxococcota**, Haliangium;<br>**Gemmatimonadota**, Gemmatimonas, f_Gemmatimonadaceae;<br>**Desulfobacterota**, Citrifermentans;<br>**Chloroflexi**, c_KD4-96, f_Roseiflexaceae, o_C0119;<br>**Bacteroidota**, Flavisolibacter;<br>**Acidobacteriota**: o_Acidobacteriales, Candidatus_Koribacter, Bryobacter, f_Acidobacteriaceae_Subgroup_1;	**Ascomycota**: Fusicolla, Alternaria, f_Nectriaceae, Trichoderma, Knufia, Acremonium, Pyrenochaeta, Pichia, Gymnoascus, o_Pleosporales, Trichocladium, Cephalotrichum, c_Archaeorhizomycetes, Didymella, Fusidium, Scutellinia, Schizothecium, o_Xylariales, Oidiodendron, Setophoma, Thelonectria, Epicoccum, Metarhizium, Stagonosporopsis;<br>**Basidiomycota**: Lentinula, Saitozyma, f_Mycenaceae, Cutaneotrichosporon, f_Thelephoraceae, f_Ceratobasidiaceae, Vishniacozyma, Apiotrichum, Wallemia;<br>**Rozellomycota**: o_Branch02, o_GS11;
pH	P	**Firmicutes**: Ruminiclostridium, Hydrogenispora, f_Christensenellaceae, Oxobacter, o_Desulfotomaculales;<br>**Proteobacteria**: Anaeromyxobacter;<br>**Myxococcota**: Ruminiclostridium;<br>**Chloroflexi**: f_JG30-KF-AS9, Ktedonobacter;<br>**Bacteroidota**: Flavisolibacter;<br>**Actinobacteriota**: Luedemannella;<br>**Acidobacteriota**: Candidatus_Solibacter, o_AKIW659;	**Ascomycota**: Aspergillus, Westerdykella, Pseudeurotium, Sagenomella, Melanocarpus, Ramophialophora, o_Eurotiales, Cercophora;<br>**Basidiomycota**: Wallemia;
	N	**Actinobacteriota**: o_Gaiellales, Streptomyces, Nocardioides, Acidothermus, f_67-14, o_Frankiales, Gaiella, o_IMCC26256, f_Nocardioidaceae, Conexibacter, c_Acidimicrobiia, Jatrophihabitans, Actinoallomurus, Mycobacterium, Solirubrobacter, Geodermatophilus, o_Frankiales, f_Geodermatophilaceae, c_Actinobacteria, Amycolatopsis, Oryzihumus;<br>**Chloroflexi**: f_JG30-KF-CM45, Sphaerobacter, c_JG30-KF-CM66, Nitrolancea, c_OLB14, f_AKYG1722;<br>**Proteobacteria**: Chujaibacter;<br>**Nitrospirota**: Nitrospira;	**Ascomycota**: Humicola, Chaetomium, o_Chaetothyriales, Papulaspora, Latorua, f_Pleosporales_fam_Incertae_sedis;
	P	**Actinobacteriota**: Amycolatopsis, Nocardioides,Gaiella, c_Acidimicrobiia, o_IMCC26256, f_Nocardioidaceae, Conexibacter, Jatrophihabitans,	**Ascomycota**: Nigrospora, Fusicolla, Acrocalymma, Neocosmospora, Trichocladium, Cephaliophora, Chaetomium, f_Nectriaceae,

F.oxysporum		o_Frankiales, Geodermatophilus, o_Gaiellales, f_67-14, Solirubrobacter, c_Actinobacteria, Knoellia, o_Frankiales, f_Geodermatophilaceae, Mycobacterium, Oryzihumus;<br>**Proteobacteria**: Reyranella, Chujaibacter, o_Elsterales, f_Xanthobacteraceae;<br>**Chloroflexi**: c_OLB14, Nitrolancea, c_JG30-KF-CM66, c_Gitt-GS-136, Sphaerobacter, f_AKYG1722;<br>**Nitrospirota**: Nitrospira;	Chaetothyriales, f_Pleosporales_fam_Incertae_sedis, Gibberella, Humicola, Echria, Schizothecium, Fusarium, Acremonium;<br>**Basidiomycota**: Ceratobasidium;
	N	**Firmicutes**: Clostridium_sensu_stricto_12, Thermincola, p_Firmicutes, p_Firmicutes, f_Hungateiclostridiaceae, f_Christensenellaceae, f_Symbiobacteraceae, Bacillus, Oxobacter, o_Desulfotomaculales, f_Limnochordaceae, Hydrogenispora, Ruminiclostridium;<br>**Actinobacteriota**: Luedemannella, Micromonospora, o_AKIW659, Geothrix;<br>**Chloroflexi**: Ktedonobacter, f_JG30-KF-AS9;<br>**Proteobacteria**: f_Rhodocyclaceae, Tepidiphilus;<br>**Myxococcota**: Anaeromyxobacter;	**Ascomycota**: Coniochaeta; Westerdykella; f_Sordariaceae; Sagenomella; p_Ascomycota; Emmonsiellopsis;<br>**Basidiomycota**: c_Agaricomycetes;<br>**Olpidiomycota**: Olpidium;<br>**Zoopagomycota**: Syncephalis;

Note: ‘c_, o_, f_’ indicated unclassified level at class, order, family level, respectively. P meant positive; N meant negative; ‘/’ meant the microbes with a significant correlation to soil environment factors were not founded.

Table S5. Standardized Indirect effects from structural equation model (SEM).

Pathways	Effect size	Percentile method 95% CI		
		Lower	Upper	P
PQQ → Available N	-0.08	-0.15	0.07	0.46
PQQ → Bacterial community	-0.43	-2.27	-0.15	0.04
PQQ → Fungal community	-0.59	-15.77	0.70	0.28
PQQ → Pathogens	-0.92	-0.97	-0.88	0.04
AP → Bacterial community	-0.21	-0.43	-0.16	0.03
AP → Fungal community	-0.35	-1.71	-.70	0.30
AP → Pathogens	-0.03	-1.02	3.27	0.94
AN → Pathogens	-0.24	-2.04	1.43	0.61
pH → Pathogens	-0.03	-1.04	2.76	0.91
